# Scientific Items

**Published:** 1879-11-20

**Authors:** 


### SCIENTIFIC ITEMS.

**Moral Dietetics.**—Dr. Bock, of Leipsic, writes as follows on the moral effect of different articles of food and drink: "The nervousness and peevishness of our times are chiefly attributable to tea and coffee; the digestive organs of confirmed coffee-drinkers are in a state of chronic derangement, which reacts on the brain, producing fretful and lachrymose moods. Fine ladies addicted to strong coffee have a characteristic temper, which I might describe as a mania for acting the persecuted saint. Chocolate is neutral in its psychic effects, and is really the most harmless of our fashionable drinks. The snappish, petulant humor of the Chinese can certainly be ascribed to their immoderate fondness for tea. Beer is brutalizing; wine impassions; whiskey infuriates, but eventually unmans. Alcoholic drinks, combined with a flesh and fat diet, totally subjugate the moral man, unless their influence be counteracted by violent exercise. But with sedentary habits they produce those unhappy flesh sponges which may be studied in metropolitan bachelor halls, but better yet in wealthy convents. The soul that may still linger in a fat Austrian abbot is functional in his body only as salt is to pork —in preventing imminent putrefaction."—*Journal of Chemistry.*

**The Mound Builders.**—A curious exhibition was made in the grain department of the Cincinnati Chamber of Commerce, being a quantity of carbonized or charred corn taken from a pit in one of the burial grounds of the mound builders near Madisonville, Ohio, a few miles distant from Cincinnati.

The pit was paved at the bottom with boulders, and contained some three or four bushels of corn, some shelled, some on cobs. The entire mass was apparently thoroughly charred. It was covered with a layer of gray ashes containing bones of animals, and still above this was a layer of clay and soil. It is supposed to have been used in some of the religious rites of the mound builders, being located in the midst of one of their large burial grounds, whence many skeletons have been removed during the past few months.

From the trees which have grown above these graves, and whose roots intersect the skeletons, there can be no doubt that the race of which this is the work must have existed here in America, fully five hundred years ago.—*Heb. Leader.*

**Alexis St. Martin.**—Alexis St. Martin, famous in physiological works for the expertments of Dr. Beaumont, is still alive, and at present a resident of St. Thomas, Joliette county, province of Quebec, Canada, and is seventy-eight years old. The wound in his stomach is never closed, and at present the opening in his side is nearly an inch in diameter. His general health appears not to have been in any way affected by the curious wound in his side, but has always been excellent. For his age he is now quite strong and hearty. He has been the father of twenty or more children, of whom four are now living. He has always been a hard worker, and never suffered from lack of digestion.—*Journal of Chemistry.*

**Origin of Plants.**—Cabbage grew wild in Siberia; buckwheat originated in Siberia; celery originated in Germany; the potato is a native of Peru; the onion originated in Egypt; tobacco is a native of South America; millet was first discovered in India; the nettle is a native of Europe; the citron is a native of Asia; oats originated in North Africa; rye came originally from Siberia; parsley was first discovered in Sardinia; the parsnip is a native of Arabia; the sun flower was brought from Peru; spinach was first cultivated in Arabia; the pear and apple are from Europe; the horse chesnut is a native of Thibet; the quince came from Island of Crete; the radish is a native of China and Japan; the pear is supposed to be of Egyptian origin; the horse radish came from the south of Europe.

**Anticipation of the Microphone.**—In her work on the "Physical Basis of Immortality," published in 1876, Antoinette Brown Blackwell said: "It remains to invent some instrument which can so retard the too rapid vibration of molecules as to bring them within the time adapted to human ears; thus we might comfortably hear plant movements carrying on the many processes of growth, and possibly we might catch the crystal music of atoms vibrating in unison with the sunbeams. Sound can be refracted by passing it through a lens which retards its motion. Such an improvement upon the stethoscope would reveal phenomena but little more marvelous than those already offered us by the telescope and microscope."—*Journal of Chemistry.*

**Gnoscopine·**—The well known English chemists, T. & H. Smith, announce the discovrry by them of a hitherto unknown alkaloid of opium, which they have named gnoscopine. This new principle is characterized by forming readily caystallizable salts, which have an acid reaction. That its salts possess this reaction, as also the fact that gnoscopine is quite insoluble in water, marks its strong resemblance to the papaverine group. Hence, also, it is easily separated from narceine, which is moderately soluble in boiling water, and freely so in alkalies. Gnoscopine when pure is in the form of long, thin white needles, having a woolly character when dried. It is soluble in 1,500 parts of cold water and melts at 238 degrees C. It is insoluble in aqueous or in spirituous solutions of caustic soda, also in mineral spirit and fusel oil, but is soluble in chloroform and bisulphide of carbon, and slightly so in benzole.—*Druggists Circular.*

**Petrolina and Petrolina Oil** are the latest aspirants for pharmaceutical honors. They are manufactured from petroleum, without the use of acids or alkalies. Petrolina is similar to and may be used for the same purposes as cosmoline and vaseline; it is the quintescence of petroleum, with all its objectional qualities removed, while all its healing and soothing properties are increased.—*Druggists Circular.*

A LAKE in Afghanistan, heretofore believed to have no outlet, has lately overflowed its banks, so that its waters have found their way into a neighboring river. This is the Ab-istada Lake, on the table-land. The discovery that it thus probably belongs to the basin of the Helmund, the great river of Western Afghanistan, is due to the recent invasion of the Cabul region by the British forces.

